# Using GRADE methodology for the development of public health guidelines for the prevention and treatment of HIV and other STIs among men who have sex with men and transgender people

**DOI:** 10.1186/1471-2458-12-386

**Published:** 2012-05-28

**Authors:** Elie A Akl, Caitlin Kennedy, Kelika Konda, Carlos F Caceres, Tara Horvath, George Ayala, Andrew Doupe, Antonio Gerbase, Charles Shey Wiysonge, Eddy R Segura, Holger J Schünemann, Ying-Ru Lo

**Affiliations:** 1 Department of Medicine, State University of New York at Buffalo, New York, USA; 2 Department of Clinical Epidemiology & Biostatistics, McMaster University, Hamilton, Canada; 3 Department of International Health, Johns Hopkins Bloomberg School of Public Health, Baltimore, USA; 4 Instituto de Estudios en Salud, Sexualidad y Desarrollo Humano, Universidad Peruana Cayetano Heredia, Lima, Peru; 5 Instituto de Estudios en Salud, Sexualidad y Desarrollo Humano, Universidad Peruana Cayetano Heredia, Lima, Peru; 6 Cochrane Review Group on HIV/AIDS, University of California, San Francisco, USA; 7 The Global Forum on MSM & HIV, Oakland, CA, USA; 8 Independent Consultant, Broadbeach Waters, Australia; 9 Department of HIV/AIDS, World Health Organization, Geneva, Switzerland; 10 Institute of Infectious Disease and Molecular Medicine & Division of Medical Microbiology, University of Cape Town, Cape Town, South Africa; 11 Universidad Peruana Cayetano Heredia, Lima, Peru

## Abstract

**Background:**

The World Health Organization (WHO) Department of HIV/AIDS led the development of public health guidelines for delivering an evidence-based, essential package of interventions for the prevention and treatment of HIV and other sexually transmitted infections (STIs) among men who have sex with men (MSM) and transgender people in the health sector in low- and middle-income countries. The objective of this paper is to review the methodological challenges faced and solutions applied during the development of the guidelines.

**Methods:**

The development of the guidelines followed the WHO guideline development process, which utilizes the GRADE approach. We identified, categorized and labeled the challenges identified in the guidelines development process and described the solutions through an interactive process of in-person and electronic communication.

**Results:**

We describe how we dealt with the following challenges: (1) heterogeneous and complex interventions; (2) paucity of trial data; (3) selecting outcomes of interest; (4) using indirect evidence; (5) integrating values and preferences; (6) considering resource use; (7) addressing social and legal barriers; (8) wording of recommendations; and (9) developing global guidelines.

**Conclusion:**

We were able to successfully apply the GRADE approach for developing recommendations for public health interventions. Applying the general principles of the approach while carefully considering specific challenges can enhance both the process and the outcome of guideline development.

## Background

The human immunodeficiency virus (HIV) epidemic is disproportionately affecting men who have sex with men (MSM) and transgender people in low- and middle-income countries [1,2], as well as in high-income countries [3]. These populations are similarly at higher risk of Chlamydia trachomatis and Neisseria gonorrhea infections relative to the general population [4]. Addressing the HIV epidemic in these specific populations at the global level is essential to control and reverse the epidemic in the general population.

The WHO Department of HIV/AIDS was charged with the development of guidelines for delivering an evidence-based, essential package of interventions for the prevention and treatment of HIV and other sexually transmitted infections (STIs) among MSM and transgender people in the health sector in low- and middle-income countries [5].

WHO’s guideline development process [6] uses the Grades of Recommendation, Assessment, Development, and Evaluation (GRADE) approach [7]. There has been substantial debate about the best grading framework in the field of public health [8-11]. Our experience developing guidelines is a useful case study providing lessons learned and insights into the use of GRADE in the development of public health guidelines. It would also inform challenges encountered in the related fields of health systems and policymaking. The objective of this paper is to review the methodological challenges faced and solutions applied during the development of these guidelines.

## Methods

### GRADE framework

GRADE presents a systematic and transparent framework for clarifying questions, determining the outcomes of interest, summarizing the evidence that addresses a question, and moving from the evidence to a recommendation or decision [12-14]. Additional file 1 and Additional file 2 respectively present GRADE definitions, categories, and factors affecting the quality of evidence and the strength of recommendation [15,16]. GRADE is currently the most widely accepted and used framework for developing guidelines. More than 50 organizations worldwide, many highly influential, have endorsed the framework ( http://www.gradeworkinggroup.org/).

### Guideline development

A core working group consisted of community representatives, content experts, systematic reviewers and a guideline methodologist. The group initially drafted specific guideline questions following the PICO format (PICO refers to Population, Intervention, Comparison, Outcomes) [17]. For each of the PICO questions, the working group conducted systematic reviews and developed GRADE evidence profiles to summarize the evidence and rate the quality of evidence [12-14] ( Additional file 3: example of an evidence profile) [7].

The core working group held three face to face working meetings in preparation for the final consensus meeting. For the final consensus meeting, the core working group developed for each PICO question a decision table summarizing judgments about: the quality of evidence, the balance of benefits and harms, values and preferences, resource use, and feasibility ( Additional file 3: example of a decision table) [12]. The final consensus meeting was held in Beijing, China in September 2010. The consensus panel consisted of community representatives, content experts, systematic reviewers, a methodologist, and national HIV/AIDS and STI program managers. Community representatives were members of the core working group and the consensus panel. The guidelines were published in June 2011 [18].

Here are the interventions addressed by the guidelines:

1. Condom use, and serosorting

2. Adult male circumcision

3. Human papilloma virus (HPV) and hepatitis A and B vaccination;

4. Periodic testing for asymptomatic STIs and STI syndromic management;

5. Behavioral interventions (individual, group and community levels);

6. HIV testing and counseling;

7. Use of outreach, social marketing and the internet to reach target constituencies;

8. Complementary mental health interventions to address substance use, and harm reduction programs among injection drug users and among transgender people who undertake gender enhancement procedures involving the injection of substances.

### Determination of the challenges

The core working group conducted discussions of methodological challenges through email communications and during the three working meetings and the final consensus meeting. We subsequently identified, categorized and labeled the identified challenges. We then finalized through an interactive process of in-person and electronic communication the solutions to these challenges along with illustrative examples from the guidelines.

We defined public health interventions as those targeting policymakers or public health professionals as well as affected populations, and are implemented in the form of programs. These include biomedical, behavioral, structural, and health system interventions. Clinical interventions target individual health consumers and their clinicians, are implemented by individuals, and consist mostly of biomedical interventions. Some biomedical interventions (e.g., screening for HIV) can be implemented both as a public health intervention (e.g., HIV screening program in an STI clinic) and as a clinical intervention (e.g., a clinician offering HIV screening to a patient based on individual risk assessment).

## Results

We have identified the following nine challenges: (1) heterogeneous and complex interventions; (2) paucity of trial data; (3) selecting outcomes of interest; (4) using indirect evidence; (5) integrating values and preferences; (6) considering resource use; (7) addressing social and legal barriers; (8) wording of recommendations; and (9) developing global guidelines. We discuss below and summarize in Table 1 each of these challenges and how we addressed them. The order of presentation is not related to the seriousness of the challenges. The first four challenges relate to the grading of the quality of evidence while the other five relate to the grading of the strength of recommendation.

**Table 1:** Summary of the challenges and applied solutions

**Challenge**	**Solution applied**
Heterogeneous and complex interventions	· We defined a priori the classification of complex interventions (e.g., classifying behavioral interventions into individual, group and community levels)
	· When interventions were combined, we considered separately each component intervention to the extent that the available evidence allowed it
Paucity of trial data	· We considered evidence for effectiveness from observational studies
	· We used cross sectional studies to derive baseline risk appropriate for low, intermediate, and high risk groups
Selecting the outcomes of interest	· We selected outcomes in a transparent and comprehensive manner, and a priori
	· We developed outcome frameworks depicting causal pathways
Using indirect evidence	· For each intervention, we made judgments about the importance of the indirectness of the population (e.g., when applying evidence from MSM population to a transgender population) and of the setting (e.g., when applying evidence from high income countries to low- and middle-income countries)
	· We downgraded the quality of evidence when indirectness was judged as serious
Integrating values and preferences	· Community representatives were involved in the development and review of the guidelines
	· We conducted a survey of community members about the values and preferences they attach to the outcomes and interventions considered in the guideline questions
Considering resource use	· We did not consider this factor in a systematic and formal way
	· For each question, experts made judgments about the implications of resource use
Addressing social and legal barriers	· We issued ‘good practice recommendations’ based on the principles of medical ethics and human rights
Wording of recommendations	· We used the term ‘conditional’ (instead of weak) for non-strong recommendation
	· We explained for each recommendation what the “conditions” for adoption are
Developing global guidelines	· The survey of values and preferences recruited participants globally.
	· We prioritized evidence of from low- and middle-income countries when available
	· The panelists prioritized the perspective of low- and middle-income countries

### Heterogeneous and complex interventions

The nature of public health interventions differs from that of most clinical interventions in a number of respects. While clinical interventions can be heterogeneous, complex and used in combination, public health interventions tend to possess these characteristics more frequently and to a larger degree [19].

We frequently identified heterogeneity in the characteristics of interventions evaluated in studies. For example, studies examining group behavioral interventions varied in the number of participants per group, in the number of sessions, and in the topics discussed (e.g., sexual risk behavior, safer sex, condom use, healthy relationships, etc.).

Our challenge was to decide whether these interventions were similar enough to consider (and meta-analyze) these studies together, and to ultimately issue one or multiple recommendations. In the case of behavioral interventions, we classified them *a priori* based on whether they were implemented at the individual, group or behavioral level. We faced similar difficulties with the reviews of sex venue-based interventions, social marketing campaigns, and Internet-based targeted interventions.

Many public health interventions are implemented in combination with other interventions because of their assumed synergistic effect. For example, counseling interventions are implemented along with HIV testing to improve understanding and behavior change. In the field of HIV, this has been recently discussed as combination HIV prevention [20,21]. Furthermore, the specific combination of interventions depends on the setting and local needs– the “know your epidemic know your current response” approach [22].

For global guidelines, it is not practical to issue recommendations for all possible combinations of interventions, and it is very unlikely that studies addressing all different combinations exist. Our approach was to consider separately each individual intervention to the extent that the available evidence allowed this. The aim was to provide a menu of effective individual interventions for policymakers to choose from according to their local conditions and need. The main limitations to this approach are potentially missing any synergistic effect, and dealing with evidence assessing combinations of interventions.

Another methodological challenge associated with the evaluation of these interventions is the risk of contamination (e.g., with information diffusion interventions) and the need for cluster randomized trials [23]. Also, the success of some of these interventions depends on the individuals implementing them (e.g., behavioral change interventions), requiring careful evaluation methods [24].

### Paucity of trial data

The scarcity of trial data and using non-trial data were a matter of debate during our guideline development process, as they have been for others involved in guideline development [25].

The evidence for all but four questions came from observational studies resulting, according to the GRADE approach, ( Additional file 1) [13] in ‘very low’ or ‘low’ quality of evidence for 12 out of 15 graded recommendations. Also, according to the GRADE approach, recommendations based on lower quality of evidence are likely to be conditional, as opposed to strong ( Additional file 2) [12]. Out of the 15 grade recommendations, 10 were graded as conditional.

Some panelists at the guidelines consensus meeting were uncomfortable with the relatively low rating of the quality of evidence. They argued that conducting randomized controlled trials (RCTs) for public health questions might be either challenging (e.g., community level implementation of a behavioral intervention), or impossible (e.g., legal interventions). Therefore, they felt that the available evidence from non-randomized studies should be rated higher [26-28].

However, the fact that the best available evidence comes (or could only come) from observational study designs does not automatically imply that these designs provide high quality evidence. Indeed, reasons for which we have less confidence in these designs relative to RCTs are valid irrespective of whether trial data is (or could be) available. In addition, observational studies could potentially provide “moderate” and even “high” quality evidence within the GRADE framework [15]. For example, the quality of evidence from cohort studies for the effectiveness of condom use among MSM was rated up to “moderate” due to a large effect size (relative risk of 0.34).

Panelists may have been uncomfortable with the low rating of the quality of evidence because it would likely lead to a weak recommendation. There was a concern that policymakers may use both “low quality evidence” (i.e., low confidence in effect estimates) and “weak recommendations” as excuses to forgo the implementation of the recommendation. Please refer to challenge (8) on how we used wording of recommendations to address these concerns.

Although GRADE stipulates that trial data provide the least-biased evidence for the effects of interventions, it does consider other types of studies. Firstly, it considers observational studies for assessing (1) the long term and rare effects of interventions, and (2) the effectiveness data in the absence of trial data, which – as discussed above – may lead to moderate or high quality evidence ( Additional file 1). It is possible that observational data could provide higher quality evidence than poorly conducted trials.

Secondly, in order to generate accurate absolute estimates of effects, GRADE calls for deriving baseline risks of outcomes from observational studies. For example, in assessing the absolute effects of behavioral interventions on HIV incidence, we derived the baseline risk from cross sectional studies appropriate for the low-, intermediate-, and high-risk groups [29,30].

Thirdly, assessing public health interventions benefits from a broad range of evidence, e.g., from the behavioral and social sciences [25]. One example is the use of process evaluation of complex interventions [31]. By capturing the experiences of participants and the details of the implementation, qualitative and quantitative studies help evaluate the different components of the intervention and investigate its contextual factors (e.g., socio-cultural factors which can act as mediators and moderators). For studies with positive results, observational data aid in assessing transferability [32]. Studies finding no effects can help in distinguishing between interventions that are inherently ineffective and those which, because of poor implementation, were not fairly evaluated [32].

Identifying process evaluation data requires additional time and resources, a specific set of skills and expertise, and searching outside the conventional biomedical databases. Moreover, this type of evidence is not readily available [33]. Indeed, we did not identify any process evaluation study for any of the RCTs considered in this guideline. This proved particularly problematic in interpreting the results of a group level behavioral intervention that unexpectedly showed increased rates of STI [34]. Subsequently, the panel was not able to reach a consensus on whether to recommend group level interventions on the basis of this one study.

### Selecting the outcomes of interest

Selecting important outcomes for each PICO question is a critical step in the development of recommendations [17], especially for public health guidelines.

The important outcomes should include all benefits and harms of the intervention. One additional important outcome that the panel considered was quality of life. In fact, it is thought that MSM are using condoms less frequently because it affects their sexual experience negatively. Another important unintended effect that the panel considered is discrimination [35-37].

**Figure 1:**
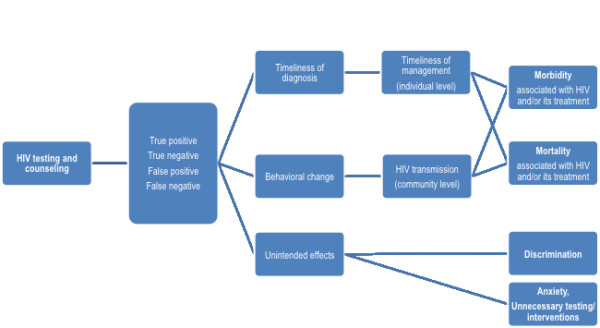
Example of an outcome framework for a testing and counseling intervention.

**Figure 2:**
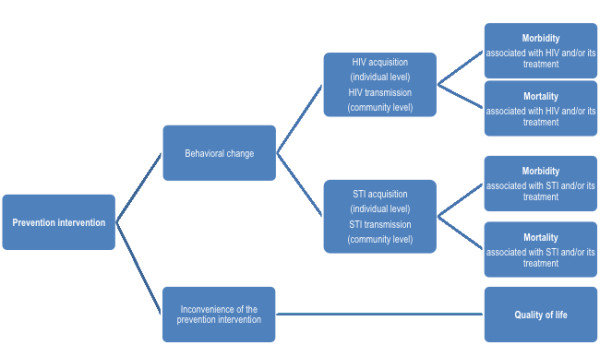
Example of an outcome framework for a prevention intervention.

The choice of important outcomes should be independent of whether or not they have been empirically assessed, while the choice of intermediate outcomes should capture those that have been empirically assessed. These outcomes should be selected in a transparent and comprehensive manner, and *a priori* (i.e., prior to reviewing the evidence). In order to achieve these goals, we developed an outcome framework for each question or group of related questions (Figures 1, 2) [38]. Each framework describes all possible pathways between the intervention and the important outcomes. 

Public health interventions can affect both individual-level and community-level outcomes. For example, an HIV testing and counseling intervention may affect the timeliness of diagnosis and treatment at the individual level, and HIV transmission at the community level (Figure 1). In both cases, the testing intervention would eventually affect both morbidity and mortality. The outcome framework helped in depicting these two distinct but convergent pathways.

The pathway for a prevention intervention has three levels of outcomes: (1) behavioral change, (2) HIV acquisition and transmission, and (3) morbidity and mortality (Figure 2). In this pathway the outcomes of utmost importance are morbidity and mortality. A conservative approach might consider these outcomes as the only important ones. Practically, this would lead to downgrading the quality of evidence associated with HIV acquisition and transmission for indirectness, leading to moderate quality evidence at best. The guideline panel made the judgment that HIV acquisition and transmission are relevant enough outcomes that downgrading the quality is not warranted. This judgment was based on the fact that a reduction in HIV transmission is highly associated with a reduction in morbidity and mortality associated with HIV infection. On the other hand, the panel made the judgment that behavioral change is an indirect outcome warranting downgrading the quality of evidence.

### Indirectness of the evidence

In addition to the indirectness of the outcome (see preceding paragraph), the panel dealt with the indirectness of the population and of the setting, a concept also known as applicability [39]. Although these guidelines address both MSM and transgender people, we did not identify direct evidence for transgender people. The panel made the judgment that the indirectness of the evidence for that population was not serious enough to warrant downgrading its quality. However, one has to acknowledge that the degree of indirectness varies across the questions. For example, the evidence is likely to be more indirect for the behavioral interventions relative to screening interventions.

As to the setting, for many PICO questions, the available evidence came solely from high-income countries. The judgment of the degree of indirectness depended on the intervention. For example, the panel judged that the evidence about the effect of condom use on HIV infection is not indirect enough when applied to low-income countries to warrant downgrading the quality of evidence. Conversely, the panel judged that the evidence about the effects of serosorting on HIV infection is indirect enough to warrant downgrading the quality of evidence, because the practice of serosorting requires regular high-quality and easily accessible HIV testing, re-testing and counseling that is frequently not available in low- and middle-income countries. Guideline panels may use more formal approaches to judging applicability [40].

### Integrating values and preferences

In the GRADE framework, the values and preferences of the target population are a major factor in determining the direction and strength of recommendations [14]. For these guidelines, the perspectives of MSM and transgender people were incorporated in a variety of ways. Firstly, community representatives were members of the core working group and the final meeting consensus panel. Secondly, two community members from a low-income and a high-income country respectively reviewed the final drafts of the guidelines. Thirdly, the WHO secretariat commissioned the Global Forum on MSM and HIV (GFMSM) to conduct a survey of both HIV positive and HIV negative MSM and transgender people from Asia, Africa and Latin America. The survey consisted of online interviews about the values and preferences community members attach to the outcomes and interventions considered in the guideline questions, the implications of the proposed guidelines, and concerns that might emerge among them from their potential implementation. The results of the survey were an integral part of the decision tables used, in some instances verbatim, at the guideline consensus meeting. The working group discussed the possibility of conducting a systematic review of studies of values and preferences relevant to the guideline question, but did not pursue this because of time and resource limitations.

The above approach of integrating values and preferences of the target population has resulted in the guidelines taking the perspective of MSM and transgender people. The advantages of that approach include improving the quality of the guidelines, and increasing their chances of acceptance and implementation by the MSM and transgender communities. One disadvantage is a potential inconsistency of some of the recommendations with differing set of values and preferences in certain settings.

### Considering resource use

Resource use is one factor that is considered in determining the direction and strength of recommendations in the GRADE framework [14]. Resource use becomes particularly important for guidelines targeting low- and middle-income countries. Indeed, the availability of resources and costs are likely to vary substantially across low- and middle -income countries. Unfortunately, the expertise and resources were not available for the panel to consider this factor in a systematic and formal way. However, the decision tables for each recommendation included a judgment about the implications of resource use. For example, condom use was seen as a relatively not resource intensive intervention. On the other hand, medical male circumcision was seen as a resource-intensive intervention, particularly in settings in which such programs are not being rolled out for the general male population. This affected the direction of the recommendation and resulted in a conditional recommendation against male circumcision in spite of low quality evidence suggesting benefits may outweigh harms.

### Addressing social and legal barriers

Discrimination, stigma, punitive laws and law enforcement practices are major barriers for MSM and transgender people in accessing health services [41-43]. This undermines the effectiveness of HIV prevention and treatment programs, particularly in low- and middle-income countries [44]. The panel felt a need to include a strong recommendation to make health services inclusive of MSM and transgender people, and more generally to ensure protective laws and regulations. However, there was no direct evidence to justify a strong recommendation, if one followed the typical GRADE approach. The resolution was to frame these recommendations as ‘good practice recommendations’ – as defined by GRADE – and base them on the principles of medical ethics and human rights [7].

Good practice recommendations are typically those in which desirable effects undoubtedly outweigh any undesirable ones so that conducting a study addressing the implicit question could not be justified [7]. Indeed, a test of whether a recommendation qualifies as a ‘good practice recommendation’ is to check whether the alternative sounds bizarre or ridiculous. GRADE suggests using ‘good practice recommendations’ for interventions that represent “necessary and standard procedures of the clinical encounter or health care system” [7].

Here are the two good practice recommendations included in the guidelines:

We recommend making health services inclusive of men who have sex with men and transgender people, based on the principles of medical ethics and the right to health.

We recommend that legislators and other government authorities establish anti-discrimination and other protective laws, derived from international human rights standards, in order inter alia to eliminate discrimination and violence faced by men who have sex with men and transgender people, and reduce their vulnerability to infection with HIV and the impacts of HIV and AIDS.

### Wording of recommendations

Guideline panels present their judgments about the quality of evidence and strength of recommendations using specific wordings of the recommendation statements and affixing grades to these statements (using a combination of letters, numbers, and symbols) [45]. Unfortunately, there is little evidence of how well various presentations are understood [45-47].

GRADE initially suggested to use the words ‘strong’ and ‘weak’ to characterize the strength of recommendations [12]. It has become clear with experience that for certain panelists ‘weak’ is not acceptable wording. Specifically, public health guideline panels worry that policymakers use this wording as an excuse to forgo the adoption of the intervention being recommended. This is in spite of the fact that a weak recommendation is intended to invite policymakers to involve their stakeholders in substantial debate in considering the intervention (as opposed to a strong recommendation intended to invite policymakers to adopt the intervention as a policy) [12]. Thus, the GRADE working group suggested alternatives to ‘weak’ such as ‘conditional’, ‘contingent’, and ‘qualified’. The core guideline working group adopted the term ‘conditional’ and the panelists were very receptive.

The potential advantage of the term ‘conditional’ is that it invites the user of the recommendation to consider the implications of that term. ‘Conditional’ can have four possible implications (or combinations of these), depending on which factor(s) affected the strength and/or direction of the recommendation ( Additional file 3). Table 2 provides the implications and examples for each of these four factors.

**Table 2:** Implications of a conditional recommendation according to the factors that affected the strength and/or direction of the recommendation

**Factor**	**Implication**	**Example**
Balance between desirable and undesirable effects	Consider whether the local incidence of the outcome of interest is high enough to tip the balance of benefits and harms in favor of implementing the intervention	*Screening for asymptomatic STIs using nucleic acid amplification tests:* “The benefits increase as the [HIV] prevalence increases. The benefits might outweigh the harms and cost if prevalence of asymptomatic urethral and rectal infections is higher than 1–2 %.”
Quality of evidence	Consider the willingness to accept the uncertainty about the effects of the intervention	*Targeted internet-based strategies:* “low quality of evidence…studies were rated down for study limitations, imprecision and indirectness.”
Values and preferences	Consider the local values and preferences	*Male circumcision*: The recommendation was conditional against in spite of the possibility of benefits outweighing harms: “participants raised questions about the relevance of circumcision in different cultural settings.”
Costs (resource allocation)	Consider the resources available and/or required locally, the local cost, and the opportunity cost given the local competing public health needs	*Community-level behavioral interventions:* “Behavioral interventions primarily require human resources for implementation; this may be a challenge in some settings. For these interventions to be successful, the necessary human resources, an enabling environment and adaptation to the local context will be necessary.”

The panel was also deliberately sensitive in wording the statement of the recommendation. While typically a recommendation would refer to the ‘use’ of a specific intervention, the guideline recommendations refer to the “offering” of an intervention. The intention was to avoid the perception of coerciveness especially in settings where MSM and transgender people are particularly marginalized and at risk of stigma, violence and abuse.

### Developing global guidelines

Developing guidelines with global scope is challenging, whether the topic is of clinical or public health nature. This may be particularly true for public health guidelines to be adopted by different countries or by different jurisdictions within a country. Indeed, the importance of the problem (and consequently the size of the effects), and the implications of an intervention (in terms of the availability of resources, costs, cost-effectiveness, acceptability, and feasibility) often vary substantially across settings.

In order to address this challenge, the survey of values and preferences recruited participants globally. We prioritized evidence of effectiveness and of incidence of outcomes from low- and middle-income countries when available. Also, the panelists were asked to prioritize perspective of low- and middle-income countries when making judgments about values and preferences, feasibility and resource use.

## Discussion

We identified and addressed a number of challenges during the development of WHO guidelines for the prevention and treatment of HIV and other STIs among MSM and transgender people in the health sector in low- and middle-income countries. While some of these challenges might be particularly relevant to public health guidelines (e.g., nature of the interventions, addressing social and legal barriers) others are common to most guidelines (e.g., adequacy of the evidence, indirectness of the evidence, wording of the recommendations). Consequently, the learning experience from this guideline may benefit other fields.

We were able to address most of these challenges (e.g., indirectness of the evidence, values and preferences) through using the principles of the GRADE approach. The use of good practice recommendations and outcome frameworks proved to be particularly useful. Successfully overcoming some of the other challenges (e.g., scarcity of evidence) requires further research in the field. Some other challenges require further methodological advancement (e.g., dealing with complex and combined interventions). The GRADE working group has been committed to and engaged in this process.

By requiring guideline developers to be systematic, explicit, and transparent in the assessment of the quality of evidence, and in determining the strength of recommendations, GRADE uncovers challenges such as those discussed in this paper. Indeed, these challenges relate to the quality of available evidence and to the complexity of guideline development process. The GRADE framework continues to evolve to address some of the issues specific to the field of public health.

One perceived limitation of the GRADE approach is its involved handling of the evidence. However, the involvement of a GRADE methodologist and the incremental learning by the members of the core working group have played a positive role. Also, using evidence profiles and decision tables to summarize the information should facilitate this process.

Finally, the GRADE approach can assist in resource mobilization. Indeed, the representative of one of the major funding bodies in the field declared at the end of the meeting that their future funding, and as a result of the rigorous process, will now take into consideration patient important outcomes such as quality of life and actual incidence of HIV, instead of reported behavioral change.

## Conclusion

We were able to successfully apply the GRADE approach for developing recommendations for public health interventions. Applying the general principles of the GRADE approach while carefully considering challenges specific to the field of public health can enhance both the process and the outcome of guideline development. Such an approach has the potential to move forward both the public health practice and research.

Finally, the process highlighted the need for more research both in the subject area (e.g., additional evaluation of interventions, studies of transgender people, low and middle-income countries) and in the methodological area (e.g., analysis of complex and combined interventions, presentation and wording of recommendations).

## Competing interests

Elie Akl, Tara Horvath, and Holger Schünemann are members of the GRADE working group.

## Authors’ contributions

EAA, CK, KK, CFC, TH, GA, AD, AG, CSW, ERS, HJS, YRL made substantial contributions to conception and design of the manuscript, and to the analysis and interpretation of data. EAA drafted the manuscript. EAA, CK, KK, CFC, TH, GA, AD, AG, CSW, ERS, HJS, YRL revise the manuscript critically for important intellectual content and have given final approval of the version to be published.

## Pre-publication history

The pre-publication history for this paper can be accessed here:

http://www.biomedcentral.com/1471-2458/12/386/prepub

## Supplementary Material

Additional file 1 **Appendix 1.** Definition, categories, and factors affecting the quality of evidence. Click here for file

Additional file 2 **Appendix 2.** Definition, categories, wording, factors affecting, and implications of the strength of recommendation. Click here for file

Additional file 3 **Appendix 3.** Examples of an evidence profile and of a decision table. Click here for file
