# Supplementary material for: Using GRADE methodology for the development of public health guidelines for the prevention and treatment of HIV and other STIs among men who have sex with men and transgender people
Source: BMC Public Health. 2012 May 28;12:386. doi: 10.1186/1471-2458-12-386 (PMC3490932; doi:10.1186/1471-2458-12-386)
Supplement: Additional file 1 — Appendix 1. Definition, categories, and factors affecting the quality of evidence. [file 1471-2458-12-386-S1.doc]

**Appendix 1:** definition, categories, and factors affecting the quality of evidence

**Definition:** The extent of our confidence that the estimate of an effect is adequate to support a particular decision or recommendation

**Categories:**

- **High**: we are very confident in the effect estimate: the true effect lies close to that of the estimate of the effect.
- **Moderate**: we are moderately confident in the effect estimate: the true effect is likely to be close to the estimate of the effect, but there is a possibility that it is substantially different.
- **Low**: we have limited confidence in the effect estimate: the true effect may be substantially different from the estimate of the effect.
- **Very low**: we have very little confidence in the effect estimate: the true effect is likely to be substantially different from the estimate of effect.

**Factors affecting quality of evidence**

| Study design | Initial grade | Grade lowered if | Grade raised if | Final grade |
| --- | --- | --- | --- | --- |
| Randomized trial 🡪 | High | Limitations of design  Inconsistency  Indirectness  Imprecision  Publication bias | Large effect  Dose response  All plausible confounding would reduce a demonstrated effect | High |
|  | Moderate |  |  | Moderate |
| Observational study 🡪 | Low |  |  | Low |
|  | Very low |  |  | Very low |
