# Supplementary material for: Using GRADE methodology for the development of public health guidelines for the prevention and treatment of HIV and other STIs among men who have sex with men and transgender people
Source: BMC Public Health. 2012 May 28;12:386. doi: 10.1186/1471-2458-12-386 (PMC3490932; doi:10.1186/1471-2458-12-386)
Supplement: Additional file 2 — Appendix 2. Definition, categories, wording, factors affecting, and implications of the strength of recommendation. [file 1471-2458-12-386-S2.doc]

**Appendix 2:** definition, categories, wording, factors affecting, and implications of the strength of recommendation

**Definition:** the extent to which we can be confident that desirable effects of an intervention outweigh undesirable effects

**Categories**

- **A strong recommendation** is one for which guideline panel is confident that the desirable effects of an intervention outweigh its undesirable effects (strong recommendation for an intervention) or that the undesirable effects of an intervention outweigh its desirable effects (strong recommendation against an intervention).
- **A conditional/weak recommendation** is one for which the desirable effects probably outweigh the undesirable effects (conditional/weak recommendation for an intervention) or undesirable effects probably outweigh the desirable effects (conditional/weak recommendation against an intervention) but appreciable uncertainty exists.

**Suggested wording**

Wording of a recommendation should offer users as many indicators as possible for understanding and interpreting the strength of recommendations.

- For strong recommendations, the GRADE working group suggests a terminology such as "we recommend..." or " should...", or a number representation with 1
- For conditional/weak recommendations, the GRADE working group suggests less definitive wording, such as "we suggest..." or " might...", or a number representation with 2

**Factors** affecting the strength of recommendation

| Factor | Explanation |
| --- | --- |
| Balance between desirable and undesirable effects | The larger the difference between the desirable and undesirable effects, the higher the likelihood that a strong recommendation is warranted. The narrower the gradient, the higher the likelihood that a conditional/weak recommendation is warranted |
| Quality of evidence | The higher the quality of evidence, the higher the likelihood that a strong recommendation is warranted |
| Values and preferences | The more values and preferences vary, or the greater the uncertainty in values and preferences, the higher the likelihood that a conditional/weak recommendation is warranted |
| Costs (resource allocation) | The higher the costs of an intervention—that is, the greater the resources consumed—the lower the likelihood that a strong recommendation is warranted |

**Implications** of strong and conditional/weak recommendations for different users of guidelines

|  | Strong | Conditional/weak |
| --- | --- | --- |
| For patients | Most individuals in this situation would want the recommended course of action and only a small proportion would not. | The majority of individuals in this situation would want the suggested course of action, but many would not. |
| For clinicians | Most individuals should receive the recommended course of action. Adherence to this recommendation according to the guideline could be used as a quality criterion or performance indicator. Formal decision aids are not likely to be needed to help individuals make decisions consistent with their values and preferences. | Recognize that different choices will be appropriate for different patients, and that you must help each patient arrive at a management decision consistent with her or his values and preferences. Decision aids may well be useful helping individuals making decisions consistent with their values and preferences. |
| For policy makers | The recommendation can be adapted as policy in most situations including for the use as performance indicators. | Policy making will require substantial debates and involvement of many stakeholders. Performance indicators would have to focus on the fact that adequate deliberation about the management options has taken place. |
