# Supplementary material for: Using GRADE methodology for the development of public health guidelines for the prevention and treatment of HIV and other STIs among men who have sex with men and transgender people
Source: BMC Public Health. 2012 May 28;12:386. doi: 10.1186/1471-2458-12-386 (PMC3490932; doi:10.1186/1471-2458-12-386)
Supplement: Additional file 3 — Appendix 3. Examples of an evidence profile and of a decision table. [file 1471-2458-12-386-S3.doc]

**Appendix 3:** Examples of an evidence profile and of a decision table

**1. Evidence Profile**

Author(s): Charles Shey Wiysonge & Muki S Shey (University of Cape Town, South Africa)
Date: 2011-03-17
Question: Should Male circumcision vs No male circumcision be used in Men who have sex with men and transgender persons?
Settings: High-income countries (16 studies), low and middle-income countries (5)
Bibliography: Wiysonge CS, Kongnyuy EJ, Navti OB, Muula AS. Male circumcision for prevention of homosexual acquisition of HIV in men. Cochrane Database of Systematic Reviews 2011 (in press).

| **Quality assessment** | | | | | | | **Summary of findings** | | | | | **Importance** |
| --- | --- | --- | --- | --- | --- | --- | --- | --- | --- | --- | --- | --- |
|  |  |  |  |  |  |  | **No of patients** | | **Effect** | | **Quality** |  |
| **No of studies** | **Design** | **Limitations** | **Inconsistency** | **Indirectness** | **Imprecision** | **Other considerations** | **Male circumcision** | **No male circumcision** | **Relative (95% CI)** | **Absolute** |  |  |
| **HIV infection (any sexual role)** | | | | | | | | | | | | |
| 20 | observational studies | no serious limitations | no serious inconsistency | no serious indirectness | no serious imprecision | none | 11753/36202 (32.5%) | 7706/28713 (26.8%) | OR 0.86 (0.70 to 1.06)^1^ | 29 fewer per 1000 (from 64 fewer to 12 more) | ⊕⊕OO LOW | CRITICAL |
| **HIV infection (mainly receptive anal sex)** | | | | | | | | | | | | |
| 3 | observational studies | no serious limitations | no serious inconsistency | no serious indirectness | no serious imprecision | reporting bias^2^ | 141/522 (27%) | 104/354 (29.4%) | OR 1.20 (0.63 to 2.29)^3^ | 39 more per 1000 (from 86 fewer to 194 more) | ⊕OOO VERY LOW | CRITICAL |
| **HIV infection (mainly insertive anal sex)** | | | | | | | | | | | | |
| 7 | observational studies | no serious limitations | no serious inconsistency | no serious indirectness | no serious imprecision | none | 158/1394 (11.3%) | 89/704 (12.6%) | OR 0.27 (0.17 to 0.44)^4^ | 89 fewer per 1000 (from 67 fewer to 102 fewer) | ⊕⊕OO LOW | CRITICAL |
| **Syphilis** | | | | | | | | | | | | |
| 6 | observational studies | no serious limitations | no serious inconsistency^5^ | no serious indirectness | serious^6^ | none | 300/23719 (1.3%) | 96/7455 (1.3%) | OR 0.96 (0.82 to 1.13) | 1 fewer per 1000 (from 2 fewer to 2 more) | ⊕OOO VERY LOW | IMPORTANT |
| **Herpes simplex virus - 1** | | | | | | | | | | | | |
| 2 | observational studies | no serious limitations | no serious inconsistency^5^ | no serious indirectness | serious^6^ | reporting bias^2^ | 748/1812 (41.3%) | 385/928 (41.5%) | OR 0.90 (0.53 to 1.52) | 25 fewer per 1000 (from 142 fewer to 104 more) | ⊕OOO VERY LOW | IMPORTANT |
| **Herpes simplex virus - 2** | | | | | | | | | | | | |
| 4 | observational studies | no serious limitations | no serious inconsistency^5^ | no serious indirectness | serious^6^ | none | 907/6698 (13.5%) | 638/3587 (17.8%) | OR 0.86 (0.62 to 1.21) | 21 fewer per 1000 (from 60 fewer to 30 more) | ⊕OOO VERY LOW | IMPORTANT |
| **Quality of life** | | | | | | | | | | | | |
| 0^7^ | no evidence available |  |  |  |  |  |  |  |  |  |  | CRITICAL |

^1^ Two studies (Reissen 2007 and Tabot 2002) reported the adjusted OR for the association between MC and HIV; with no corresponding count data. The 2 studies had a total of 869 participants with complete data on MC and HIV status. The total number of participants is therefore 65,784; and not 64,915.
^2^ Only 3 of the 20 studies reported (separate) data for MSM who play predominantly or only the receptive role in anal sex (possibility of publication bias); rated down by 1.
^3^ The Sanchez 2007 study (with 906 participants who self-identified as mainly receptive) reported the adjusted OR for the association between MC and HIV; with no corresponding count data.The total number of participants is therefore 1,782; and not 876.
^4^ The Sanchez 2007 study (with 1931 participants who self-identified as mainly insertive) reported the adjusted OR for the association between MC and HIV; with no corresponding count data.The total number of participants is therefore 4,029; and not 2,098.
^5^ Low heterogeneity: I-square = 0%.
^6^ The 95% confidence intervals include both values suggesting benefit and values suggesting harm.
^7^ Not reported

**2. Decision table**

Should male circumcision be used for prevention of HIV and other STIs among MSM and transgender people?

| **Factor** | **Explanation / Evidence** | **Judgment** |
| --- | --- | --- |
| Quality of Evidence | No RCTs in MSM to study effect of adult male circumcision. Existing observational evidence is downgraded for design, heterogeneity, and imprecision. | Very low |
| Balance of Benefits vs. Harms | Current evidence suggests a potential benefit of adult male circumcision in terms of both HIV (OR 0.86, 95%CI 0.70-1.06 for all MSM irrespective of sexual role; OR 0.27, 95%CI 0.17-0.44 for MSM who practice insertive anal sex) and a non-significant positive effect on other STIs (OR range 0.86-0.96).  Harms have not been studied but potentially include surgical complications, pain, and stigma (if circumcision offered only to MSM). | Benefits potentially outweigh harms |
| Values and Preferences | *Per the Values and Acceptability preliminary report:* “With regard to adult male circumcision as an HIV prevention strategy, participants raised questions about relevance of circumcision in different cultural settings and adequacy to protect from HIV infection, and relevance to transgender people. Some felt the focus should be on education regarding sexual health rather than circumcision.” | Values and preferences generally against male circumcision |
| Resource Use | Significant resources needed for male circumcision in settings where it is not a standard intervention. | Significant concern |
| Feasibility | Significant concerns with feasibility of implementing a surgical intervention, especially within an already stigmatized population. | Significant concern |
